# Life-Threatening Endocrinological Immune-Related Adverse Events of Immune Checkpoint Inhibitor Therapy

**DOI:** 10.3390/cancers15245786

**Published:** 2023-12-10

**Authors:** Aleksandra Basek, Grzegorz K. Jakubiak, Grzegorz Cieślar, Agata Stanek

**Affiliations:** 1 Student Research Group, Department and Clinic of Internal Medicine, Angiology, and Physical Medicine, Faculty of Medical Sciences in Zabrze, Medical University of Silesia, Batorego 15 St., 41-902 Bytom, Poland; basek.aleksandra@interia.pl; 2 Department and Clinic of Internal Medicine, Angiology, and Physical Medicine, Faculty of Medical Sciences in Zabrze, Medical University of Silesia, Batorego 15 St., 41-902 Bytom, Poland; cieslar1@tlen.pl (G.C.); astanek@tlen.pl (A.S.)

**Keywords:** cancer immunotherapy, immune checkpoint inhibitor, immune-related adverse events, adrenal crisis, thyroid storm, diabetic ketoacidosis, severe hypocalcaemia

## Abstract

**Simple Summary:**

Malignant neoplasms are currently one of the main causes of morbidity and mortality worldwide, posing a major public health challenge. However, recent advances in cancer biology and immunity research have led to the development of immunotherapy, which is now used in routine clinical practice in cancer treatment. Along with the increasing number of patients treated with immunotherapy, a wider spectrum of side effects, called immune-related adverse events, have been brought to light. Most of them have a mild or moderate manifestation. However, in rare cases of life-threatening symptoms, proper and rapid management is of utmost importance. In this review, we focus on life-threatening endocrine side effects of immunotherapy to provide information on the symptoms, diagnostics, and management strategies described in the literature to date.

**Abstract:**

Malignant neoplasms are currently one of the leading causes of morbidity and mortality worldwide, posing a major public health challenge. However, recent advances in research in cancer biology and immunity have led to the development of immunotherapy, which is now used on an everyday basis in cancer treatment in addition to surgical treatment, classical cytostatics, and radiotherapy. The efficacy of immunotherapy has promoted the great popularity of this treatment among patients, as well as significant research interest. The increasing number of patients being treated with immunotherapy not only reassures physicians of the efficacy of this technique but also shows the wide spectrum of side effects of this therapy, which has not been considered before. Immune-related adverse events may affect many systems and organs, such as digestive, cardiovascular, respiratory, skin, or endocrine organs. Most complications have a mild or moderate course, but there are life-threatening manifestations that are essential to be aware of because if they are not properly diagnosed and treated on time, they can have fatal consequences. The purpose of this paper was to present the results of a literature review on the current state of knowledge on life-threatening endocrine side effects (such as adrenal crisis, thyroid storm, myxoedema crisis, diabetic ketoacidosis, and severe hypocalcaemia) of immune checkpoint inhibitors to provide information on symptoms, diagnostics, and management strategies.

## 1. Introduction

Malignant neoplasms are currently one of the leading causes of morbidity and mortality worldwide [1], along with cardiovascular diseases [2,3]. The global cancer burden is expected to increase by 47% from 2020 to 2040 [4]. The results of scientific studies from different countries show that malignant neoplasms are a major public health challenge worldwide [5,6,7]. The problem of greatest importance is tumours located in such locations as the gastrointestinal tract [8], pancreas [9], liver [10], lungs [11], prostate [12], breast [13], or reproductive organs [14].

The treatment of malignant tumours includes three main pillars: surgical treatment, radiotherapy, and pharmacological treatment [15]. Until recently, the pharmacotherapy of malignant tumours relied primarily on classical cytostatic drugs, which were not specific to cancer cells because they acted on the basic mechanisms involved in the control of the cell cycle, which function similarly in all dividing cells. Such treatment is burdensome and is associated with numerous side effects, which are partly due to the inhibition of the cell cycle and partly due to specific mechanisms typical for some substances (e.g., the cardiotoxicity of anthracyclines) [16].

Advances in more than a decade of research on cancer immunosurveillance and the tumour microenvironment have constantly led to a better understanding of the impact of the immune system on malignancy [17]. The realised ability of cancer cells to escape the antitumour response and even create immune suppression in their environment encouraged us to seek novel therapeutic strategies regarding immune escape from tumours [18,19]. Currently, due to the significant development of cell biology, immunology, and molecular biology, as well as the development of knowledge about the cellular mechanisms of carcinogenesis in particular types of cancer, molecularly targeted substances are increasingly being used [20]. Although this type of therapy is much better tolerated, it is not devoid of side effects that develop in a different mechanism and proceed differently than in the case of classic cytostatics, often in a less predictable way. As the body of literature on adverse events of cancer immunotherapy is constantly expanding, new conditions are still being reported, teaching physicians to be on alert with patients receiving novel therapies [21].

The purpose of this paper was to present the results of a literature review about the most essential information on the current state of knowledge about the dysfunction of the endocrine glands as a side effect of cancer immunotherapy, which has life-threatening potential if left undiagnosed.

## 2. Cancer Immunotherapy

Under physiological conditions, molecules present on the surface of T-lymphocytes called immune checkpoints are key factors in maintaining immune homeostasis and preventing autoimmunity. Some checkpoints mediate stimulatory signals and others inhibit T-cell activity. Regarding tumour immunotherapy, we are especially focused on two inhibitory checkpoints: cytotoxic T-lymphocyte-associated protein 4 (CTLA-4) and programmed death protein 1 (PD-1). CTLA-4 interacts with costimulatory molecules CD80 and CD86 in antigen-presenting cells (APCs), preventing CD80/86 binding to CD28 and thus leading to the activation of signals. This interaction causes the production of regulatory cytokines, the inhibition of conventional T cells, and the inhibitory action of regulatory T cells. The PD-1 checkpoint binds to unique programmed death ligand 1 (PD-L1), present in many cells including lymphoid, endothelial, thyroid, muscle, and hepatocyte cells, and programmed death ligand 2 (PD-L2), which is found mainly in APCs. This interaction of PD-1 with specific ligands activates the intracellular inhibitory pathway that leads to T-cell exhaustion and the development of regulatory T-cell action [22,23].

**Figure 1:**
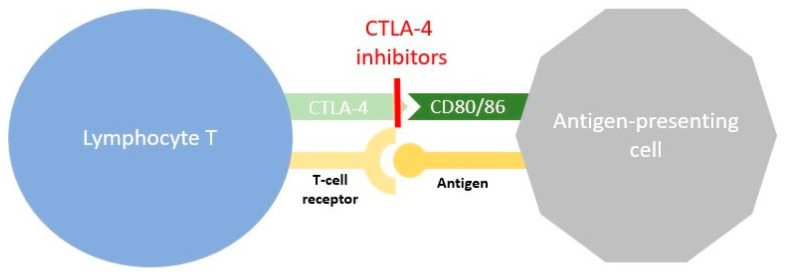
Mechanism of CTLA-4 blockade therapy. The tumour antigen in antigen-presenting cells binds to the T-cell receptor, causing an activation pathway; however, the binding of CTLA-4 with CD80 or CD86 in APC suppresses lymphocyte T activity. CTLA-4 inhibitors block the interaction between these proteins and allow the antitumour response [22,24].

**Figure 2:**
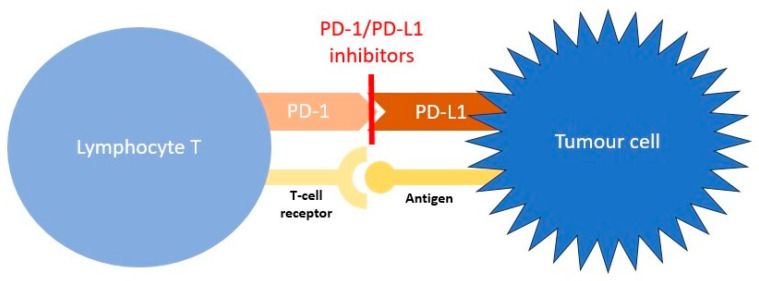
Mechanism of PD-1/PD-L1 blockade therapy. The tumour cell antigen binds to the T-cell receptor, causing an activation pathway; however, the binding of PD-1 with PD-L1 in the tumour cell suppresses lymphocyte T activity. PD-1/PD-L1 inhibitors block the interaction between these proteins and allow the antitumour response [22,32].

In the case of cancer, this immune homeostasis can be dysregulated, and the natural antitumour activity is suppressed. Malignant tumour cells develop an immune resistance mechanism that allows them to escape the host response and grow. Immune checkpoint blockade was first shown to improve antitumour activity in mice by Leach et al. [24] and the positive effect on overall survival during anti-CTLA-4 treatment was shown in many subsequent trials [25,26,27] that led to US Food and Drug Administration (FDA) approval. PD-L1 was found in many human cancer cells, which may be the reason why tumour cells evade immunity and diseases progress [28]. Furthermore, the expression of PD-1 in tumor infiltrating lymphocytes (TILs) CD8+ was found to be higher than in normal tissue T-cell infiltrates, which is the answer to its impaired function [29]. Some cancers may even develop adaptive immune resistance and express PD-L1 in response to inflammatory signals produced by an active antitumour immune response [30,31]. In turn, this opened the door to the evolution of PD-1 and PD-L1 blockade therapy. Contrary to CTLA-4 action on early T-cell activation, PD-1 affects the response of T cells at the effector stage in the tumour microenvironment. Cancer-induced suppression of TILs could be broken by blocking the PD-1/PD-L1 axis, which restores T-cell action against cancer [32]. Again, in many clinical trials, the effectiveness of PD-1/PD-L1 blockade therapy has been demonstrated [33,34,35,36,37]. The mechanisms of CTLA-4 and PD-1 blockade therapies are presented in Figure 1 and Figure 2, respectively.

The search for new targets for cancer immunotherapy is the subject of current research. T-cell immunoreceptor with immunoglobulin and ITIM domain (TIGIT) is one of the most promising new targets for immune checkpoint inhibitors (ICIs) [38]. TIGIT is an inhibitory receptor expressed on lymphocytes, which interacts with CD155 expressed on APCs or tumour cells to down-regulate T-cell and natural-killer (NK) cell functions. TIGIT is considered to be a key inhibitor of antitumour responses [39]. Monoclonal antibodies directed against TIGIT are the subject of clinical trials in numerous solid tumours and haematological malignancies [40,41].

To date, there are eleven ICIs approved by the FDA: two CTLA-4 inhibitors (ipilimumab and tremelimumab), five PD-1 inhibitors (pembrolizumab, nivolumab, cemiplimab, dostarlimab, and toripalimab), three PD-L1 inhibitors (atezolizumab, durvalumab, and avelumab), and one lymphocyte-activation gene 3 (LAG-3) inhibitor (relatlimab) [42]. The recommendation list for ICI use in therapy is constantly expanding and the number of patients eligible for ICI treatment is increasing, making it one of the most promising therapies in clinics at present.

However, new challenges have arisen for clinics with the systematic FDA approval of monoclonal antibodies directed against CTLA-4, PD-1, and PD-L1 since 2011. The list of recommended therapies for the use of ICIs is constantly expanding, and the number of patients included in drug prescription programs is increasing, making the use of ICIs one of the most promising therapies today. However, this has also brought multiple adverse events related to these drugs to light. Designed to block the immune checkpoints, they are not directed strictly toward the tumour-associated T lymphocytes, and so also affect immune responses in healthy tissues. This leads to side effects called immune-related adverse events (irAEs), which are most frequently associated with the skin, colon, endocrine organs, liver, and lungs. Some of these irAEs can occur with severe manifestations, although relatively rare, such as cardiovascular or neurologic toxicities [43].

Fatal consequences of ICI therapy vary depending on the regimen. With CTLA-4-inhibitor therapy, colitis, hepatitis, and pneumonitis are predominant, and in anti-PD-1/PD-L1 regimens, pneumonitis, hepatitis, colitis, neurological events, and myocarditis are relatively common. In the case of combination therapy, deaths are most often due to colitis, myocarditis, hepatitis, pneumonitis, and myositis; fatal endocrine toxic effects represent 5.5% of deaths, secondary to ICI toxicity [44].

## 3. Adrenal Crisis Due to Central or Primary Adrenal Insufficiency

Adrenal insufficiency during ICI treatment is mainly related to central disorder. Hypophysitis is one of the most common endocrine irAEs and occurs mainly in patients treated with CTLA-4 inhibitors or combined ICI regimens. The observed incidence of hypophysitis varies from 1.8 to 5.6% for CTLA-4 blockade and from 7.7 to 10.5% for combination therapy, while in the case of PD-1 inhibitors, it ranges from 0.3 to 1.1% and <0.1% [45,46]. The pathophysiology of this irAE is not yet completely understood; however, CTLA-4 antigen was found in pituitary endocrine cells, with the expression level varying between people due to single-nucleotide polymorphism. CTLA-4 blockade leads to the formation of antibodies against pituitary cells and a site-specific immune response that causes necrosis and fibrosis primarily in the anterior lobe. Pituitary cells are damaged as a result of the activity of the complement system and the cascade of pro-inflammatory cytokines [47,48]. It seems to mostly affect males over sixty years of age and is two to five times more frequent than in women, contrary to many autoimmune diseases [49]. Patients usually present different vague and mild-intensity complaints, such as headache, fatigue, weakness, confusion, labile moods, anorexia, nausea, or vomiting. Sometimes, although rarely, pituitary enlargement, which is a sensitive and specific indicator of hypophysitis in patients treated with ipilimumab [50], causes mass effect symptoms such as unusual headaches or visual impairment, directing clinicians to more specific diagnostics [43,51]. The most common manifestation of hypophysitis is adrenal insufficiency, but it may also present with central hypothyroidism, diabetes insipidus, and hypogonadism [52] with symptoms adequate for the disorder. Hypophysitis was found to occur after a median time of 8.4 weeks after the first administration of ipilimumab, and the cumulative effect did not appear to affect the incidence [50]. Unfortunately, adrenal insufficiency secondary to hypophysitis appears to be irreversible [53,54].

Primary adrenal insufficiency (PAI) is much less common. The body of literature concerning this condition consists almost entirely of case reports. The WHO VigiBase analysis prepared in 2019 identified only 451 cases of primary adrenal insufficiency among the 50,108 cases of irAEs between 2008 and 2018. Moreover, there were only 45 definite PAI cases, while others were possible. The analysis carried out by Grouthier et al. indicated that most cases of ICI-induced PAI affect men and are found during the seventh decade of life. Furthermore, the time range to the onset of PAI was 6 to 576 days after the first dose of ICI, highlighting the need to remain alert throughout the treatment period and suspect PAI in cases of fatigue, asthenia, nausea, hypotension, hyponatremia, or hyperkalaemia [55]. Another study reported that the incidence of this irAE is 0.7% for any grade of PAI and 0.2% for severe PAI [46]. In turn, the review by de Filette et al. showed that the predicted incidence of PAI is 1.4%, 1.3%, 2.0%, and 0.8% in patients treated with ipilimumab, tremelimumab, nivolumab, and pembrolizumab, respectively. For combination therapy of ipilimumab with nivolumab, the incidence of PAI was 5.2% and 7.6% for ipilimumab with pembrolizumab [56]. The underlying pathophysiological mechanism may be adrenalitis with hypermetabolic, symmetrically and smoothly enlarged adrenal glands [57], with the presence of autoantibodies against 21-hydroxylase, but potentially may be reversible [58,59].

Min et al. presented an interesting case report of a 56-year-old woman with metastatic melanoma who developed central adrenal insufficiency coexisting with primary adrenal insufficiency after ipilimumab therapy [60].

Central and primary adrenal insufficiency may turn out to be a severe and life-threatening condition and lead to adrenal crisis, if not diagnosed and managed in time. In the case of hypotension, nausea, vomiting, confusion, hyponatremia or hyperkalaemia, it is recommended to obtain tests for adrenal function, including morning adrenocorticotropin (ACTH) and a cortisol or cosyntropin stimulation test, as well as a basic metabolic panel. In the case of a high blood level of ACTH and a low blood level of cortisol, PAI should be suspected. In this case, it is recommended to consider adrenal imaging tests to exclude metastasis or haemorrhage. When a low cortisol concentration coexists with a low or inappropriately normal ACTH concentration, it suggests a central disorder. Suspicion of hypopituitarism should lead to the extension of the diagnostics to the thyroid axis (thyrotropin (TSH), free thyroxine (fT_4_), and free triiodothyronine (fT_3_)), the gonadal axis (luteinizing hormone (LH), follicle-stimulating hormone (FSH), and testosterone levels in males or oestrogen levels in premenopausal females), and the growth hormone axis (insulin-like growth factor 1 (IGF-1) and glucagon stimulation test), and pituitary magnetic resonance imaging (MRI) should be considered. However, in the case of severe symptoms, it is highly recommended to administer corticosteroids prior to the results of laboratory tests. The withdrawal of ICI therapy is also required. In all cases, endocrinological consultation is recommended. Long-term treatment consists of the replacement of hormones in proper doses, with regard to starting corticosteroids several days before thyroid hormone administration. Importantly, all patients with adrenal insufficiency should wear an alert bracelet, which may be helpful in the case of an emergency [22,45,49,52,61,62,63,64].

The choice of glucocorticoid dose depends on the specific clinical situation. Generally, it is recommended to start physiologic steroid replacement hydrocortisone at a dose of approximately 10 mg/m^2^ [64]. If an adrenal crisis occurs, treatment should follow the general guidelines. The typical treatment regimen for adults includes a high dose of intravenous hydrocortisone (100 mg bolus, then 200 mg daily as a continuous infusion), as well as fluid therapy, thromboprophylaxis, and mineralocorticoid supplementation (fludrocortisone), which should be initiated at the stage at which the daily dose of hydrocortisone is reduced to less than 50 mg [65].

## 4. Thyroid Storm and Myxoedema Crisis

Thyroid irAEs include common hypothyroidism, rare thyrotoxicosis, painless thyroiditis, and a life-threatening thyroid storm. Thyroid dysfunction may concern a central disorder, which was described above, and a primary disorder. Primary hypothyroidism is characterised by a high blood TSH level, as well as low to normal fT_4_ and fT_3_ blood levels. In hypothyroidism caused by central dysfunction, the TSH level is low to mid-normal, which involves a low fT_4_ level [66]. Primary thyroid disorder is one of the most common ICI-induced endocrinopathies, whereas hypothyroidism is the most common thyroid dysfunction after ICI therapy. Any grade of hypothyroidism is reported to occur in 5.6% of patients, with severe hypothyroidism in up to 0.2% of patients treated with anti-PD-1 [67]. According to a systematic review and meta-analysis performed by Almutairi et al., the incidence of all-grade hypothyroidism was 7.0–8.3%, and the incidence of all-grade hyperthyroidism was 3.0–3.4%. Severe hypothyroidism was not observed in any patients treated with ipilimumab, nivolumab, pembrolizumab, and the combined therapy of ipilimumab and pembrolizumab, while severe hypothyroidism occurred in 0.08% of patients treated with the combined therapy of ipilimumab with nivolumab. The incidence of severe hyperthyroidism was 0.1% for ipilimumab, 1.31% for ipilimumab with pembrolizumab, and 0.66% for ipilimumab with nivolumab, and no cases were found for combined therapy of pembrolizumab and nivolumab [68]. Other thyroid irAEs, such as autoimmune thyroiditis, are rare and their incidence is difficult to evaluate. The highest risk seems to be associated with women, younger patients, and the obese. Additionally, Caucasians and Hispanics are more prone to hypothyroidism, while African Americans suffer more often from thyrotoxicosis [69]. The median time to the onset of thyroid toxicity from the first dose could be different depending on the type of ICI and ranges between 113 days for combination therapy and 190 days for PD-1 therapy [54]. Hyperthyroidism has been reported to occur earlier than hypothyroidism [70].

The mechanism of ICI-induced thyroid disorders is still unclear. In the study by Osario et al., among ten patients who developed thyroid dysfunction, antithyroglobulin or antimicrosomal antibodies were found in eight patients after pembrolizumab administration [70]. Another study reported ten cases of thyroiditis after anti-PD-1 treatment. Six patients developed first transient thyrotoxicosis with subsequent hypothyroidism, whereas four patients developed hypothyroidism without the thyrotoxic phase, but in all of these patients, antithyroid antibodies were found [71]. Iyer et al. also found thyroid peroxidase and thyroglobulin antibodies in 44.7% and 33% of patients after ICI therapy, respectively [72]. However, pathways mediated by T-cells, natural killer cells, and monocytes may also participate in thyroid dysfunction during anti-PD-1 therapy, independently of antibodies [73]. Interestingly, in the positron emission tomography scan, most of the patients affected by thyroid irAEs showed a diffuse increase in 18-fludeoxyglucose uptake, which may be a better biomarker for thyroid irAEs, while thyroid antibodies may reflect the severity and increased risk of the need for hormone replacement [74]. One study found that in addition to the increase in titres of thyroid antibodies, some cytokines are also associated with thyroid irAEs. Higher levels of cytokines such as IL-1β, IL-2, and GM-CSF and lower levels of IL-8, G-CSF, and MCP-1, which indicate increased Th1/Th2 balance, may be related to the development of thyroid irAEs [75].

An interesting relationship was observed in the retrospective study performed by Kotwal et al. Patients treated with atezolizumab and avelumab who developed thyroid irAEs had longer overall survival and lower mortality [74]. A similar finding was shown in the retrospective cohort study of patients treated with nivolumab. Those who developed thyroid dysfunction had significantly longer median overall survival than patients without thyroid irAEs. However, this may be inconclusive in malignant melanoma [76].

The rare condition of thyroid eye disease consistent with Graves’ ophthalmopathy comprising eye pain, proptosis, conjunctival redness, periorbital oedema, ophthalmoplegia, and swelling of the extraocular muscles in an MRI is associated with CTLA-4 polymorphism [77] and is correlated with ICI therapy, even if the patients remain euthyroid [78,79,80].

Thyrotoxicosis is characterised primarily by tachycardia and weight loss, but also by heat intolerance, tremors, anxiety, emotional lability, hyperdefecation, oligo/amenorrhoea in women, and erectile dysfunction in men. Hypothyroidism manifests itself in weight gain, fatigue, cold intolerance, depression, weakness, dry skin, alopecia, puffiness, constipation, bradycardia, and delayed relaxation of the tendon reflexes [81]. In reported cases of thyroid storms, patients presented anxiety, high body temperature (up to 40.3 Celsius degrees), tachycardia, atrial fibrillation, nausea and vomiting, and high blood pressure. In all cases of ICI-induced thyrotoxicosis evaluated, TSH was low and thyroid hormone levels were markedly increased [82,83,84].

A myxoedema crisis is a rare adverse effect of ICI therapy. Only a few case reports describe this condition in the literature. A 53-year-old woman, treated with nivolumab due to metastatic squamous cell carcinoma of the lung, presented with slurred speech, progressive diffuse facial, periorbital, and tongue swelling, weakness, fatigue, forgetfulness, depression, constipation, dyspnoea, a slow voice, cold intolerance, and dry skin. Laboratory tests revealed remarkably higher TSH levels and undetectable fT_4_, consistent with a myxoedema crisis. Moreover, elevated creatine kinase suggested myopathy due to hypothyroidism [85]. McDonald et al. presented an interesting case of a woman with metastatic melanoma, treated with ipilimumab and nivolumab, who developed painless thyroiditis as an irAE with hyperthyroidism followed by hypothyroidism. She started oral levothyroxine supplementation and continued immunotherapy. After a few weeks, she presented with abdominal pain and profuse diarrhoea, but there was also fatigue, generalised weakness, hypotension, confusion, lethargy, and periorbital oedema. Blood tests identified profound hypothyroidism, despite oral substitution for thyroxine, and she was diagnosed with myxoedema coma. Further investigation showed immunotherapy-related enteritis with oral thyroxine malabsorption, which was the cause of the presented hypothyroidism [86]. Another reported case of myxoedema crisis occurred in a 70-year-old male with metastatic lung adenocarcinoma treated with pembrolizumab. In the routine monitoring of thyroid function during oncological treatment, decreased fT_4_ and increased TSH were found, and levothyroxine was administered. However, he experienced hematemesis from multiple oesophageal and pre-pyloric ulcers, as further investigation showed. After a few days, the patient was found to be unresponsive, hypothermic, with severe oedema, and without a detectable pulse. After returning to spontaneous circulation, he was treated in the intensive care unit for hypothermia and multiple organ failure, including respiratory and circulatory failure. Due to the results of the blood tests of TSH and fT_4_, the patient was diagnosed with myxoedema coma and intravenous administration of levothyroxine was immediately initiated [87]. In all the cases of myxoedema crisis presented here, intravenous levothyroxine was initiated in conjunction with intravenous steroids to prevent adrenal insufficiency.

Some reports in the literature also describe myopathy and rhabdomyolysis during hypothyroidism after ICI treatment [88,89,90,91].

To prevent thyroid-related AEs, it is recommended to evaluate thyroid function tests (TSH, fT_3_, and fT_4_) regularly during the ICI treatment. Most oncological societies suggest evaluating baseline thyroid tests before starting treatment and then monitoring every four to six weeks during therapy. The ESMO guidelines indicate that during anti-CTLA-4 therapy (including combination with anti-PD-1), thyroid function should be monitored every cycle and after four cycles every four to six weeks, but in the case of anti-PD-1/anti-PD-L1 therapy, it should be measured every cycle for the first three months and every second cycle thereafter. Hypothyroidism requires the test for antithyroperoxidase antibodies. If TSH is low or normal and fT_4_ is decreased, the morning cortisol level should be checked to exclude hypopituitarism with adrenal insufficiency. Low TSH with elevated fT_4_ may indicate Grave’s disease and requires the evaluation of anti-TSH receptor antibodies, antithyroperoxidase antibodies, and thyroid uptake scans. Hyperthyroidism, even subclinical, may also be present at the beginning of painful thyroiditis, which often precedes hypothyroidism. This requires regular thyroid monitoring and the consideration of steroid administration [52,61,62,63,64].

Treatment of ICI-related thyroid irAEs is described in a comprehensive way in accessible recommendations. For asymptomatic hypothyroidism with TSH less than 10 mIU/L, only careful observation and thyroid function testing are necessary every four to six weeks. However, in the case of a TSH level greater than 10 mIU/L, the administration of levothyroxine should be considered. When hypothyroidism is clinically obvious, thyroid hormone supplementation with monitoring is recommended to bring TSH to within the reference range. Furthermore, antithyroperoxidase antibody testing is recommended here. If symptoms are of moderate intensity and do not disturb activities of daily living, there is no need to perform ICI therapy in every case; however, it is recommended to consider endocrinological consultation. Severe symptoms with medically significant or even life-threatening consequences, preventing the patient from performing activities of daily living, always require ICI therapy and thyroid hormone supplementation. Sometimes hospitalization and intravenous therapy may be essential. In all cases of overt hypothyroidism, concomitant adrenal insufficiency must be excluded prior to thyroid administration. Hyperthyroidism requires frequent (every four to six weeks or even two to three weeks) thyroid function monitoring because of the possible transition to hypothyroidism in the case of thyroiditis. In cases of asymptomatic hyperthyroidism or those with mild symptoms, the patient can continue ICI therapy with close follow-up and monitoring, and usually no treatment is required. In moderate-grade hyperthyroidism, ICI therapy and endocrinological consultation should be considered, as well as methimazole or propylthiouracil administration. Furthermore, these patients may benefit from β-blocker (e.g., propranolol, atenolol) administration. In the case of persistent hyperthyroidism, additional tests should be performed to rule out Graves’ disease, including for the TSH receptor antibody and the thyroid-stimulating immunoglobulin antibody, as well as the thyroid iodine uptake scan or the technetium 99 m thyroid scan. In hyperthyroidism, it is also recommended to check for anti-TPO. Severe or life-threatening symptoms of hyperthyroidism require stopping ICI therapy and treatment in the hospital according to standard guidelines depending on previous diagnostics. It is important to closely monitor TSH and fT_4_ and to evaluate symptoms in the case of thyroiditis to catch the conversion to the hypothyroid phase and introduce thyroid hormone supplementation. ICI therapy may be reintroduced when symptoms and laboratory results improve to at least moderate intensity [45,49,52,61,62,63,64].

## 5. Diabetic Ketoacidosis

Diabetes mellitus (DM) is a rare irAE reported in 0.9–1.9% of patients treated with ICIs in retrospective studies. Patients receiving CTLA-4 monotherapy are significantly less likely to develop ICI-related DM than patients who receive PD-1/PD-L1 monotherapy. The risk of combination therapy, which carries a significant risk of other irAEs compared with monotherapies, has not been fully described in the case of ICI-related DM [92]. In a systemic review and meta-analysis of 101 articles, which also considered clinical trials and prospective studies, DM was also mainly related to PD-1/PD-L1, with an incidence of 2.0% (95% CI, 0.7–5.8) for nivolumab and 0.4% (95% CI, 0.2–1.3) for pembrolizumab. In this study, no DM cases on anti-CTLA-4 therapy were observed [56]. According to a systemic review, DM occurs after 4.5 cycles of ICIs on average, and earlier for combination therapy (2.7 cycles), but cases of early-onset DM were also observed in all treatment regimens. ICI-related DM seems to be predominant in males and occurs at a mean age of 61. However, it is probably due to the epidemiology of the cancers most frequently treated with these drugs. Among 91 cases of DM reviewed by de Filette et al., up to 64 (71%) developed diabetic ketoacidosis (DKA), which is a life-threatening condition [93].

The mechanism of ICI-related DM is still poorly understood but is considered similar to type 1 DM in some aspects—for example, the loss of pancreatic β-cell function due to an autoimmune process. Based on studies on mice, PD-1/PD-L1 axis blockade results in the appearance of activated islet-specific T cells, which initiate and mediate autoimmune β-cell destruction [94,95]. In humans, PD-L1 is expressed on β-cells in cases of inflammation and the presence of inflammatory mediators like interferon γ. In the case of type 1 DM, this up-regulation leads to a delay in the destruction of pancreatic islets. However, in cancer, which is also an inflammatory state, blocking PD-1/PD-L1 may precipitate autoimmunity and lead to DM [96]. Interestingly, a relatively high number of patients suffer the sudden onset of ICI-induced DM, similar to the onset of type 1 DM. Some authors even compare this with the fulminant type of DM, which is more common in Asia than in Europe and is defined as hyperglycaemia and ketoacidosis contrasted with a nearly normal or moderately increased percentage of glycated haemoglobin (HbA1c) [92,97]. In the analysis of the medical history of twenty-seven patients with DM after ICI therapy, the acute presentation of symptoms was observed in 59% of patients with an average glucose level of 653 mg/dL and a rapid loss of β-cell function evidenced by undetectable C-peptide levels. Moreover, blood activities of lipase or amylase were increased, which may suggest the ongoing inflammatory process in the pancreas. Islet autoantibodies were present in 40% of patients, and the predominant haplotype was HLA-DR4 (76%) [98]. The systematic review performed by Filette et al. showed the onset of DKA in 71% of cases, with a median glycaemic index of 565 mg/dL and low levels of C peptides. Furthermore, lipase levels were elevated in 52% of cases, and 53% had at least one positive islet autoantibody. A total of 61% of cases had genotypes susceptible to type 1 DM with predominant HLA-DR4 [93]. In the case of a series study of ten patients who developed ICI-related DM performed by Tsang et al., all participants had high glucose levels (over 360 mg/dL) and inappropriately low C-peptide levels (median 0.35 nmol/L). However, only two patients had positive diabetes-associated autoantibodies tests and three patients had a risk haplotype of type 1 DM [99]. Apparently, the presence of islet autoantibodies is not a rule in ICI-induced DM, in contrast with classical type 1 DM, in which the vast majority of patients are positive for one or more of these antibodies. A case report and literature review study conducted by Gauci et al. showed that islet autoantibodies may be present before ICI treatment in non-diabetic patients and, when exposed to ICI therapy, may precipitate the onset of DM. Therefore, the predictive value of these antibodies requires further study, especially since ICI-induced DM has a sudden onset in many cases [97].

The clinical presentation of DM may comprise polyuria, polydipsia, and weight loss. The development of the above symptoms should prompt further diagnostics for possible DM. According to the National Comprehensive Cancer Network (NCCN) guidelines, in the case of new-onset fasting glucose above 200 mg/dL or random blood glucose above 250 mg/dL or above 250 mg/dL with a history of DM, new-onset ICI-related DM must be considered [63]. However, ICI-related DM presents most frequently as DKA with nausea or vomiting, abdominal pain, hyperventilation, and even coma. This onset shares many clinical features with fulminant DM mentioned previously, such as the rapid onset of acidosis, high plasma glucose with relatively low HbA1c, and a low serum C-peptide level [45]. Suspicion of DKA should lead to a test of serum glucose level, blood gases and pH, urine or serum ketones, and a basic metabolic panel [45,63]. Also, anti-islet antibodies should be tested to distinguish autoimmunologic DM from type 2 DM [49,64]. Since DKA is a life-threatening disease, urgent management consistent with the current guidelines is essential, including intravenous fluid supplementation, insulin therapy, and potassium replacement. Patients require hourly monitoring of their glucose and ketone levels, as well as blood gas checks [100]. In the case of ICI-induced DM without DKA, ICI therapy is recommended when fasting glucose is greater than 250 mg/dL, but in all cases of ketoacidosis, ICI therapy should be stopped; however, after the correction of metabolic disorders, a return to ICI treatment should be considered [64]. To prevent the development of ICI-related DM and DKA, it is recommended to monitor the patient for hyperglycaemia at the baseline and every cycle during induction for twelve weeks and then every three to six weeks [52].

## 6. Severe Hypocalcaemia

Severe hypocalcaemia as a complication of ICI treatment is a rare and potentially life-threatening irAE, secondary to hypoparathyroidism. Due to limited data on this irAE, its incidence is hard to estimate. According to a retrospective study conducted by Nalluru et al., among 178 patients included in the analysis, a case of true hypocalcaemia (corrected for albumin) during treatment with ICIs was observed in only one patient (0.56%). However, this study had its limitations due to the uneven distribution of ICI type, the retrospective nature of the study, and the high incidence of cases lost to follow-up [101]. In the retrospective study performed by Bai et al., VigiBase data were collected between January 2011 and March 2019, and 11 cases of ICI-induced hypoparathyroidism were found among the 6089 patients analysed (0.18%) [102]. In a similar study, data were collected from the first quarter of 2014 to the first quarter of 2019 in the FDA Adverse Event Reporting System (FAERS) database. Among the total of 6260 cases of endocrine irAEs, 18 cases presented with hypoparathyroidism (0.29%) [103]. Despite the disparity of available reports, hypoparathyroidism due to ICI therapy seems to be exceedingly rare. Usually, it develops between one and twelve months from the beginning of therapy and is most often associated with anti-PD-1 or combination therapy [102,104]. In reported cases, hypoparathyroidism was observed after treatment with pembrolizumab [105,106,107], durvalumab [108], nivolumab [109], and a combination of ipilimumab and nivolumab [110,111,112]. About 77.8% of hypoparathyroidism cases present as life-threatening severe hypocalcaemia and require hospitalization [113].

The pathophysiology of ICI-related hypoparathyroidism is still unclear. However, it is supposed to have an autoimmune basis since, in many reported cases, antibodies against the calcium-sensing receptor (CaSR) were present [105,109,110] with negative tests for other antibodies [109,110]. However, compared with control patients after the same therapy, CaSR autoantibody levels were similar, suggesting that detected antibodies were not pathogenic [112]. The probable mechanism may consist of a T-cell reaction, activated through immune checkpoint blockade, to autoantibodies that cause inflammation in parathyroids.

Autoimmune hypoparathyroidism may occur in isolation or be part of autoimmune polyendocrine syndrome 1 (APS1) [114]. This occurrence has been identified in differentiation diagnosis serum tests for antibodies against cytokines, including interferons (INF-α2 and IFN-ω), which are specific to specific autoantibodies of APS1 [115] and NACHT leucine-rich repeat protein 5 (NALP5), which are common in hypoparathyroidism in APS1 [116]. However, hypoparathyroidism has not yet been described as a part of APS1 in connection with ICI treatment.

ICI-induced hypoparathyroidism manifests itself mainly in general fatigue and weakness, but in some of the described case reports, other symptoms occurred such as nausea and vomiting, paraesthesia, dizziness, ataxia, abdominal cramps, or disbalance. Based on physical examination, some patients had positive Chvostek and Trousseau signs [105,108]. Also, ECG showed prolonged QT in some patients [107,109,111,112,117]. In laboratory tests, low or undetectable serum levels of parathyroid hormone (PTH), hypocalcaemia, and hyperphosphatemia are remarkable [118] and were present in all reported cases of hypoparathyroidism. Furthermore, vitamin D deficiency is also common.

Importantly, the evaluation of magnesium levels is essential to differentiate hypocalcaemia due to hypoparathyroidism from the increased bone absorption of calcium in thyrotoxicosis [119].

Treatment of severe hypocalcaemia includes urgent intravenous administration of 8.5% calcium gluconate. Also, the supplementation of active vitamin D should be started [119]. There is no evidence for the effect of glucocorticoid intake, and it is not recommended to use high doses of glucocorticoids [120]. In reported cases, the symptoms of hypocalcaemia resolved after the treatment. However, the patients had to continue to supplement with calcium and vitamin D, as parathyroid function did not recover in cases of follow-up [105,110,111,117]. ICI therapy should be stopped until general conditions are stable [119].

## 7. Conclusions

Since the first approval of immune checkpoint inhibitors for clinics by the FDA in 2011, this group of samples has been implemented in oncologic treatment used in daily practice, contributing to an improved prognosis of many cancers and a longer life expectancy of cancer patients. On the other hand, the rapid use of ICI therapy has pointed to the need to gain insights into the adverse events related to these novel therapies.

The awareness of irAEs is growing constantly with the number of subjects receiving ICI therapy, as the case reports, reviews, and guidelines develop each year. The known adverse effects of ICIs affect many systems, including the skin, cardiovascular, musculoskeletal, lung, gastrointestinal, and endocrine systems. Most irAEs have mild or moderate courses; however, some may be severe and potentially life-threatening if not properly diagnosed and treated on time. Awareness of these states and symptoms and the ability to correlate them with ICI therapy is imperative for clinicians to properly treat new conditions. However, it should be emphasised that, according to the current state of knowledge, severe endocrine side effects are rare during treatment with ICIs. According to Wang et al., fatality rates in ICI-induced hypophysitis and adrenal insufficiency have been estimated at 2% and 3.7%, appropriately [44].

Table 1 summarises the most important findings of our literature review in relation to individual endocrine side effects of cancer immunotherapy in the context of symptomatology, diagnosis, and treatment.

## Figures and Tables

**Table 1:** Summary of life-threatening irAEs: symptomatology, diagnosis, and management.

Adverse Event	Main Signs and Symptoms	Basic Assessment	Management	References
Adrenal crisis	Hypotension, nausea, vomiting, confusion as well as signs and symptoms of hyponatremia or hyperkalaemia	(1) Adrenocorticotropin and cortisol blood level (2) Sodium and potassium (3) Basic metabolic panel	(1) Steroids (hydrocortisone)	[45,49,52,118]
Thyroid storm	Tachycardia, high body temperature, anxiety, high blood pressure, nausea, and vomiting	(1) Thyrotropin, free thyroxin (2) Antithyrotropin receptor antibodies	(1) Thyrostatic (methimazole or propylthiouracil) (2) β-blocker (e.g., propranolol, atenolol) (3) Steroids in some cases	[45,49,52,61,62,118]
Myxoedema crisis	Swelling, bradycardia, weakness, fatigue, hypothermia, depression, constipation, dyspnoea, slow voice, dry skin	(1) Thyrotropin, free thyroxin	(1) Thyroid hormone supplementation	[45,49,52,61,62,118]
Diabetic ketoacidosis	Nausea or vomiting, abdominal pain, hyperventilation, coma	(1) Serum glucose level (2) Blood gases and pH (3) Urine or serum ketones (4) Basic metabolic panel (5) Anti-islet antibodies (6) Glycated haemoglobin (7) C-peptide	(1) Fluid supplementation (2) Insulin therapy (3) Potassium replacement	[45,49,52,118]
Severe hypocalcaemia	Nausea and vomiting, paraesthesia, dizziness, ataxia, abdominal cramps or disbalance, positive Chvostek and Trousseau signs	(1) Parathormone (2) Calcium and phosphorus (3) Vitamin D3 (4) ECG (possible prolonged QT)	(1) Treatment with 8.5% calcium gluconate (2) Vitamin D3 supplementation	[45,108,111,119]

## Data Availability

The data presented in this study are available in this article.
